# Principles and Practices of Community Engagement in AI for Population Health: Formative Qualitative Study of the AI for Diabetes Prediction and Prevention Project

**DOI:** 10.2196/69497

**Published:** 2025-10-02

**Authors:** Ibukun-Oluwa Omolade Abejirinde, Ijeoma Uchenna Itanyi, Kathy Kornas, Remziye Zaim, Shion Guha, Victoria Chui, Lorraine Lipscombe, Laura C Rosella, James Shaw

**Affiliations:** 1 Institute for Better Health, Trillium Health Partners, 2nd floor, 2085 Hurontario Street, Mississauga, ON, L5A 4G1, Canada, 1 437-247-0596; 2 Dalla Lana School of Public Health, University of Toronto, Toronto, ON, Canada; 3 Research and Innovation Institute, Women's College Hospital, Toronto, ON, Canada; 4 Faculty of Information, University of Toronto, Toronto, ON, Canada; 5 Department of Computer Science, University of Toronto, Toronto, ON, Canada; 6 Department of Medicine, Temerty Faculty of Medicine, University of Toronto, Toronto, ON, Canada; 7 Institute for Clinical Evaluative Sciences, Toronto, ON, Canada; 8 Department of Laboratory Medicine and Pathobiology, Temerty Faculty of Medicine, University of Toronto, Toronto, ON, Canada; 9 Department of Physical Therapy, Temerty Faculty of Medicine, University of Toronto, Toronto, ON, Canada

**Keywords:** artificial intelligence, Canada, community engagement, diabetes, patient engagement

## Abstract

**Background:**

Preventing diabetes is a priority for governments and health systems worldwide. Artificial intelligence (AI) has the potential to inform prevention and planning. However, there is little guidance on how patients, caregivers, and communities are engaged in AI life cycle stages.

**Objective:**

This formative qualitative study aimed to identify principles for meaningful community engagement. The goal was to support the responsible use of machine learning models in diabetes prevention and management.

**Methods:**

We conducted a literature scan on how AI or digital health initiatives have engaged patients and communities. A participatory workshop was then organized with patients, caregivers, community organizations, clinicians, and policymakers. In the workshop, we identified and ranked guiding principles for community engagement in AI for population health. We also outlined key considerations for implementing these principles.

**Results:**

We identified 10 principles for patient and community engagement in AI for health care from 6 papers and developed a conceptual framework for community engagement on AI. A total of 30 workshop participants discussed and ranked the top 6 principles: trust, equity, accountability, transparency, codesign, and value alignment. Participants noted that embedding community engagement in the AI life cycle requires inclusivity and diversity. Additionally, implementers should leverage existing resources and adopt a centralized approach to AI decision-making.

**Conclusions:**

Our study offers useful insights for community-focused AI deployment that centers the values of patients and communities. The identified principles can guide meaningful engagement on the use of AI in health systems, while future research can operationalize the conceptual framework.

## Introduction

Artificial intelligence (AI) has significant potential to improve health outcomes and health care effectiveness [1,2]. In particular, machine learning, a subset of AI, has demonstrated strong predictive capabilities in disease risk assessments for cardiovascular diseases, cancers, Alzheimer disease, and diabetes [3,4]. Despite such potential, the translation of AI to solve population health concerns remains slow due to multiple barriers, including implementation challenges, limited validation, data uncertainties, and social acceptability concerns [5,6]. Current research on the use of AI in health care primarily emphasizes macro-level governance and technical optimization, with less attention given to patient and community perspectives [7]. Although patients and communities are directly impacted by AI-informed health care decisions, their engagement in its design and deployment is limited [8,9]. Enhanced social acceptability, achieved through meaningful community engagement, can improve AI uptake, yet guidance on systematically engaging communities in AI deployment remains scarce [9].

With an estimated prevalence of 783 million cases by 2045, type 2 diabetes (T2D) is a public health issue globally [10]. In Canada, diabetes affects approximately 11.9 million people and significantly impacts health care expenditure [11]. Structural barriers, such as socioeconomic disparities, limited access to health care and healthy foods, and restrictions posed by built environments, continue to impede population-wide diabetes prevention efforts [12,13]. Addressing these barriers requires scalable, inclusive strategies, potentially supported by AI-driven solutions that are collaboratively designed and well-implemented [14]. Members of our research team previously used routine health administrative data of patients accessing the publicly funded health system in 1 Canadian province to develop and validate 2 machine learning models. These models predict the risk of T2D onset and diabetes-related complications and have demonstrated robust predictive accuracy across diverse populations. The details of the model development and validation are reported elsewhere, and they show consistent calibration across sociodemographic subgroups by sex, ethnicity, immigration status, and material deprivation [15,16]. In 2023, we received funding from the Canadian Institute for Advanced Research to establish the AI for Diabetes Prediction and Prevention (AI4DPP) Solution Network. The goal of the network is to deploy both machine learning models by (1) identifying implementation barriers, (2) developing a visual analytics dashboard of population-level diabetes risk, (3) creating a governance framework for responsible AI deployment, and (4) evaluating the use of the model and dashboard at a population level.

Several studies highlight the role of patient and public engagement in shaping more equitable and context-specific interventions [17-19]. The value of multi-stakeholder engagement in enhancing accountability and trustworthiness of AI systems has also been established [20]. However, there is comparatively limited guidance on how patient and public engagement occurs across the AI life cycle. Some initiatives have begun to address this gap. A recent scoping review on AI and machine learning applications in health care identified 21 papers (of 10,880 searched) that mentioned community involvement at any stage of the AI life cycle [9]. Another example is the Artificial Intelligence/Machine Learning Consortium to Advance Health Equity and Researcher Diversity in the United States to understand community perspectives on AI and its impact on health equity [21]. Participants from that study emphasized the need for context-sensitive, hyperlocal approaches to AI design and deployment.

This study contributes to the limited body of research on how to meaningfully engage communities in the deployment of AI tools for public health. We applied insights from a literature scan to inform a participatory workshop involving professionals and people with lived and living experience of T2D.

## Methods

We used a formative qualitative study design to explore the principles and associated practices of community engagement in the implementation of population-level AI tools for T2D prevention and management.

### Study Setting

The AI4DPP project is being implemented in Peel region, Ontario [22], which has a high prevalence of T2D, particularly among South Asian, Asian, Arab, African, and Hispanic populations [23]. In 2015, the incidence of diabetes in Peel was 1192/100,000, reflecting an increase of 182% within 2 decades [24].

Our team works with health system practitioners, decision-makers, community organizations, and people with lived and living experience of T2D in Peel region. This partnered approach aims to ensure that the 2 machine learning models can be implemented in a way that is responsive to local needs [25] when applied at a population level. The AI4DPP Solution Network hosts a biannual full-day meeting for patient and public stakeholders in Peel region to share project updates. The May 2024 in-person edition of the network meeting served as the setting for the participatory workshop we conducted for this study.

### Ethical Considerations

This study was reviewed by Trillium Health Partners Research Ethics Board and was determined to not qualify as human participant research (reference #247). The AI4DPP study also underwent ethics review and approval by the Health Sciences Research Ethics Board at the University of Toronto, Dalla Lana School of Public Health (protocol number: 46174). When invitations to the network meeting were sent out, we notified participants that anonymized handwritten notes for research purposes would be taken during the workshop and that no participant would be identified or quoted by name in the analyses or outputs. Invitees were requested to contact the research team if they had questions or would like to opt out ahead of the meeting. Verbal group consent and confidentiality of discussions were established at the beginning of the meeting. Community representatives and people with lived and living experience were each given a CAD $150 (US $107.60) gift card as honorarium after the meeting. We also reimbursed their parking and transportation costs, where applicable.

### Data Collection and Analysis

We conducted a literature scan [26] to identify strategies for engaging community organizations and people with lived and living experience on AI for health care. This was followed by a 90-minute participatory workshop at the AI4DPP Solution Network meeting.

#### Literature Scan

We conducted a literature scan to identify and build upon existing knowledge on patient and public engagement in AI for population health. The goal was not to conduct a comprehensive review but to inform the design of the workshop. At least 2 members of the research team reviewed recent literature in the PubMed database using random iterative combinations of the following search terms with Boolean operators (AND, OR): “community engagement,” “patient engagement,” “artificial intelligence,” “machine learning,” “digital technology,” and “health.” No date limits or restrictions on the number of results were applied, but priority was given to papers published within the last 5 years that described frameworks or strategies for patient and public engagement on AI in health.

From an initial batch of 27 identified papers, we screened papers by title, abstract, and full-text review. This yielded 6 relevant papers that captured guidance on processes and strategies that support patient and community engagement. We synthesized review findings using a narrative approach informed by qualitative content analysis [27]. To do this, we reviewed full texts, identifying key themes related to community and patient engagement and grouped findings into principles. Where papers reported specific frameworks or approaches used to inform their work, we took note of these. We ultimately used the AI life cycle as a framework for conceptualizing where (ie, stage of life cycle) and how (ie, principles) patient and public engagement was occurring. The AI life cycle has been described by different authors with varying degrees of detail and language, but with several points of overlap in the core ideas they capture [21,28,29]. In summary, the AI life cycle can be described to include 5 broad stages: (1) *define* (ie, definition of the health problem for which an AI tool will be developed), (2) *design and test* (ie, development, validation, and testing of the AI model including assessing the model for bias and fairness), (3) *deploy* (ie, implement the AI model for use in the real world), (4) *evaluate* (ie, assess the performance of the AI model in the real world, and identify areas for improvement), and (5) *improve* (ie, update the AI model using real-world data and evaluation findings). This analysis resulted in the development of a conceptual framework on the interplay between the AI life cycle and levels of public participation (see the Results section).

#### Participatory Workshop

##### Overview

A participatory multistakeholder workshop was conducted during the May 2024 AI4DPP network meeting. Other activities conducted during the full-day meeting that are not reported in this paper included a presentation on AI adoption in health care and a session to elicit feedback on the analytics dashboard we were designing as part of the overall project. The decision to use a participatory approach was informed by its effectiveness in eliciting diverse stakeholder perspectives, fostering equity in participant contributions, and promoting collective decision-making through structured activities [30].

##### Recruitment

Identification of participants used both convenient and purposive recruitment methods. Due to the capacity limits of the venue, we planned for approximately 40 attendees. First, we extended invitations to individuals who had attended the inaugural AI4DPP Solution Network meeting in November 2023. Because people with lived and living experience of T2D and community representation had been limited at that event, we purposefully reached out to community organizations in Peel who provide T2D prevention and management services, leveraged our existing relationships with Peel Public Health and connections from prior community-based research projects.

To recruit people with lived and living experience, we used a previously successful approach wherein we collaborated with the Patient and Family Partners (PFP) unit of a large hospital system in Peel. Through the PFP coordinator, the project overview and meeting details were shared with PFP members with an invitation to the workshop. We conducted 3 virtual briefing sessions to accommodate varying schedules for 6 individuals who expressed interest. Briefing sessions covered meeting agenda, logistics (eg, accessibility, dietary needs, parking), honoraria, and transportation reimbursement. Informed consent was obtained during these sessions, and participants were encouraged to ask questions.

##### Workshop Facilitation

IOOA and IUI, who have expertise in qualitative research and community engagement methods, jointly facilitated the workshop. Facilitation employed a modified format of the nominal group technique (NGT)—an effective method for enabling active discussions within group settings [31,32] and managing equitable participation [33]. This was an important consideration in our study where the different professional roles of participants introduced power differentials. The workshop included the following:

1. A presentation on participatory approaches to community engagement, findings from the literature scan, and the framework for community engagement on AI which was developed by the research team based on the literature scan.
2. In line with nominal group technique, perspectives on meaningful community engagement were elicited through silent idea generation by way of individual reflections and note-taking following question prompts such as “*Who is the community?*” and “*How should meaningful community engagement in the AI4DPP project be realized?*”*.*
3. Ranking exercises on individuals’ mobile phones or laptops using an online poll created in Mentimeter (2024). Mentimeter is a web-based tool that enables real-time audience participation and generates ranked output based on weighted responses. Each participant was asked to rank their top 6 principles from the 10 identified in the literature scan. The decision to select 6 principles was because we deemed this number feasible to manage—providing a breadth of options but easy to recall and keep discussions focused. Before ranking, participants were asked to suggest principles they felt were relevant but not captured in the literature findings. We facilitated a round-robin discussion after the results of the ranking exercise were revealed.
4. A walk-around exercise wherein participants offered suggestions on how the prioritized principles can be put into action (ie, operationalized). Insights on best practices for each of the 6 engagement principles were captured on sticky notes. Participants also noted additional considerations and potential challenges to realizing each principle.

We invited participants to consider their responses to the discussion prompts not only within the AI4DPP project, but for other conceivable population-level AI health applications. The meeting was not audio-recorded, but 2 trained note-takers documented discussions throughout the day. At the start of the meeting, attendees were reminded that the activities and outputs of the meeting would be used for research purposes. Sociodemographic data were not collected, as it was not essential to workshop objectives and might have discouraged participation. However, we recognize that participants brought additional forms of diversity including racial, cultural, and socioeconomic experiences, which may have shaped their perspectives.

##### Workshop Data Analysis

After the workshop, 2 members of the research team with experience in qualitative methodology collated data from sticky notes, flipcharts, and meeting notes. Analysis was guided by a thematic approach where we integrated both deductive and inductive strategies. Deductive coding was guided by the principles identified from the literature scan, which served as a reference framework. The data were manually organized, clustering similar contributions and developing summary memos to refine the framing of each principle and associated practices for operationalizing them. In parallel, we conducted inductive coding to allow new themes and insights to emerge directly from the workshop data. This iterative process supported convergence across participant perspectives, leading to the final set of principles and practices reported in the Results section. Our analysis was not geared to inform theoretical knowledge but to highlight practical insights related to meaningful community engagement in AI for population health.

## Results

### Overview

First, we present findings from the preworkshop activities—literature scan and the conceptual framework for community engagement on AI developed by the research team. Next, workshop findings are presented under 3 subheadings: participants’ perspectives on community engagement on AI, key principles for community engagement on AI, and practical considerations for operationalizing the key principles. A total of 30 people attended the workshop: 6 people with lived and living experience (patients and caregivers), 2 community organization representatives, 3 senior leaders from Peel Public Health, 4 health care providers, 12 AI4DPP team members (including 4 trainees), and 3 representatives from the project funder, the Canadian Institute for Advanced Research.

### Literature Findings: AI and Community Engagement

Synthesis across the 6 papers resulted in a compilation of 10 community engagement principles for AI: (1) trust, (2) power-sharing, (3) empowerment, (4) value alignment, (5) equity, (6) codesign, (7) transparency, (8) education, (9) early engagement, and (10) accountability. Practices identified in the literature for engaging patients and communities in AI and technology interventions included a need for early and meaningful engagement of patients and communities, ensuring diversity of participants [34-36] and adopting transparent and bidirectional communication where the community feels empowered and able to participate in decision-making [36]. Patient education was emphasized as being crucial to meaningful engagement to inform patient-centered design and build trust [34,36]. The papers drew from diverse approaches, particularly community-based participatory research and citizen science.

The International Association for Public Participation spectrum of engagement—a framework that defines levels of public engagement starting from being informed to being consulted, being involved, collaborating, and finally, empowering—the highest level of engagement [36,37], was explicitly adopted or referenced in most of the 6 papers. At the first level of the International Association for Public Participation spectrum*—inform*—patients and community organizations are informed about an AI-related health project including the benefits, risks, and potential impact. At the second level, *consult*, patients’ and community organizations’ perspectives on the feasibility and utility of the project are solicited. At *involve,* the third stage, project teams should be working with patients and community organizations to consider and address any concerns related to the AI intervention, and at the *collaborate* stage, patients and community organizations should be active decision-makers regarding the project. When at the *empower* level, patients and community organizations should ideally take the lead in setting priorities and making decisions on the project. Collectively, these insights from the literature captured in Table 1 informed the discussion prompts for the workshop.

**Table 1.** Overview of selected literature on community engagement for artificial intelligence (AI) and digital health.

Reference	Summary
Adus et al [34]	Qualitative study to understand patient perspectives on being engaged in the development of AI. Findings emphasized early patient involvement in problem identification, diverse recruitment strategies, and multi-modal engagement methods. A framework for effective patient engagement in AI development is proposed, emphasizing the importance of addressing health inequities and ensuring patient-centered design [34].
Pillai et al [36]	The authors advocate for a paradigm shift in health care natural language processing to address how the perspectives of patients and community members are frequently overlooked. They propose a community-based natural language processing (CBNLP) framework, inspired by the principles of community-based participatory research. The framework outlines 5 levels of community engagement that align with the International Association for Public Participation (IAP2) levels of public engagement [36].
Moodley and Beyer [38]	This study makes a call for an Afro-centric community engagement model for genomic banking based on Ubuntu, an African philosophy. By emphasizing interconnectedness and communal well-being, Ubuntu can significantly enhance community engagement practices. Authors propose an 8-step framework called the TRUCE model to guide effective and ethically sound community engagement in genomic biobanking. Community consultation and co-ownership of knowledge production throughout the research process are recommended, similar to the CBNLP framework [38].
Richardson et al [39]	This paper emphasizes the ethical imperative of understanding and incorporating patient views to ensure AI technologies align with patient needs and values. A conceptual implementation-evaluation framework for AI that highlights the interplay of patient experiences, health systems, and technology is proposed [39].
O’Connor et al [40]	The paper presents synthesis from 19 qualitative studies on the barriers and facilitators impacting patient and public engagement with digital health interventions. Findings are presented in the Digital Health Engagement Model, which focuses on 4 main factors that influence individuals’ willingness to engage with and enroll in digital health interventions—perceived value, assessed quality, engagement approach, and ability to actively use the intervention. Digital health developers and implementers are encouraged to prioritize and invest in patient-centered approaches to foster trust and ensure equitable access to emerging health care technologies [40].
Barony Sanchez et al [35]	Using the Virtual Community of Patients and Citizens Partners (COMVIP) project as an example, this study calls for patient and citizen engagement as an essential component of digital health technologies. Beyond tokenism, implementers are encouraged to actively involve patients and citizens as partners in the technology co-creation process using iterative and inclusive approaches. Authors argue that a multistage cocreation process involving patients and communities will ensure that technologies are tailored to the needs and preferences of diverse patient populations, leading to a more inclusive and equitable digital health ecosystem [35].

### Conceptual Framework for Community Engagement on AI

#### Overview

**Figure 1.**
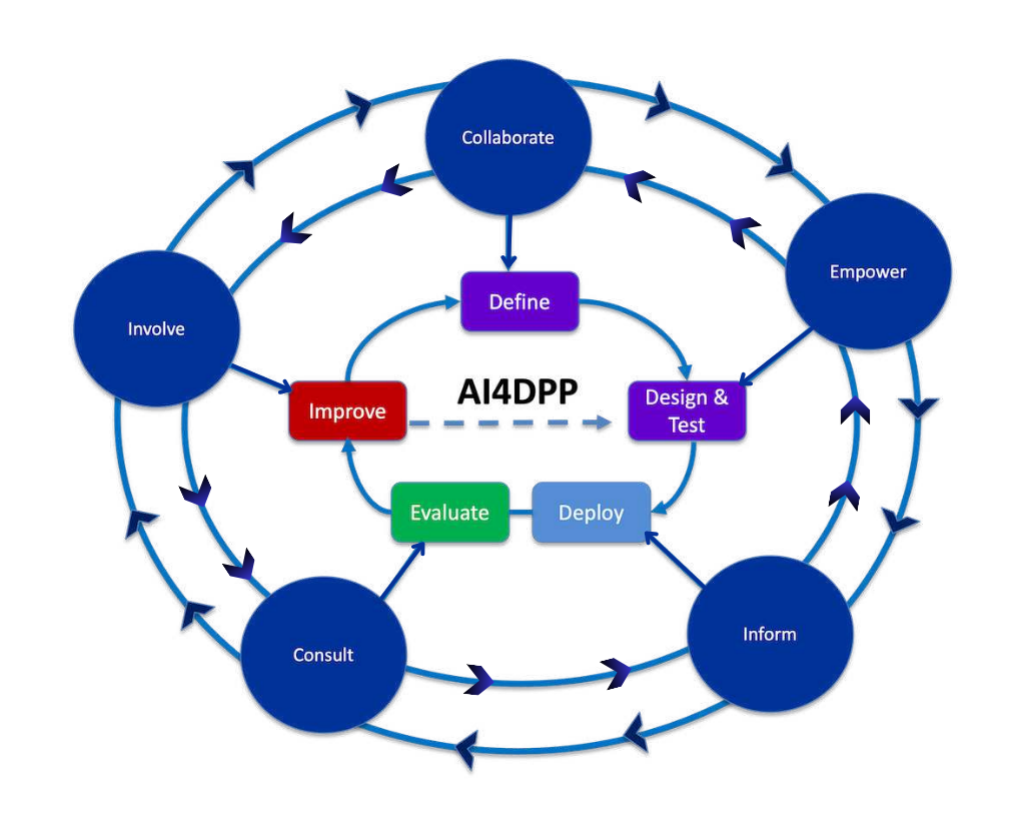
Conceptual framework of interplay between community engagement and artificial intelligence (AI) life cycle. All levels of public participation may be employed at each stage of the AI life cycle in a cyclical and iterative fashion. Learnings from the “improve” stage of the life cycle feedback into a continuous process of redesign and testing. AI4DPP: AI for Diabetes Prediction and Prevention.

Since the IAP2 spectrum of public participation emerged as the most frequently referenced framework for community engagement, the research team conceived that for community engagement to be integrated into the use of AI tools for public health, the 5 cyclical stages of the AI life cycle should be integrated with the 5 levels of public participation. AI projects that seek to meaningfully engage patients or community organizations at any stage of the AI life cycle can do so at any of the IAP2 levels. This was captured in a conceptual framework developed by the research team (Figure 1) that shows the superimposed and interconnected relationship between the AI life cycle (define, design and test, deploy, evaluate, and improve) and IAP2 levels of public participation (involve, collaborate, empower, consult, and inform). The AI4DPP project served as the first case study through which this conceptual framework was explored (Figure 1).

#### Participants’ Perspectives on Meaningful Community Engagement on AI

Workshop participants expressed that meaningful community engagement should be inclusive and representative of the target end beneficiary and should adopt diverse accessible formats of communication (eg, newsletters, arts-based methods, reports, infographics). Participants suggested that culturally appropriate strategies for engagement should be tailored to the different population groups, especially in a setting as diverse as Peel. Emphasis was placed on leveraging existing resources and strengths in the community such as faith-based organizations or community peer workers to facilitate building trust, community participation, and ownership of AI-driven innovations. A notable community asset, which was highlighted during the group discussion, was the community health ambassadors’ (known in other contexts as community health workers or cultural brokers) model. Community ambassadors play critical health promotion roles and are best suited to reach structurally marginalized groups and facilitate community engagement [41].

Additionally, participants spoke to the sustainability of engagement activities, recommending a departure from project-specific initiatives to a centralized regional model in Peel involving other sectors such as pharmaceutical companies and municipal leaders. There was encouragement to consider using the AI4DPP project as an exemplar to demonstrate the feasibility of community engagement in deploying AI models, which can be adapted for other health-related AI applications and geographic locations. Patient and community organization representatives emphasized that engagement must occur consistently and on the terms of the community, not of the AI intervention.

#### Key Principles of Community Engagement on AI for Population Health

When meeting attendees provided feedback on the conceptual framework (Figure 1) and the 10 engagement principles identified in the literature scan, there was agreement that community engagement can be initiated at any stage of the AI life cycle, starting from any level of the engagement spectrum. The starting point for engagement was said to depend on the extent to which previous projects and relationships had set the stage for a collaborative model of engagement.

No new principles were suggested to be added to the 10 identified ones. Rather, it was decided that 2 of the principles—education and early engagement—were already captured by other principles. Thus, workshop participants collectively decided to focus the discussion on the other 8 principles. Participants emphasized the importance of involving community organization representatives, but also directly involving people with lived and living experience—ensuring diverse representation of age, sex, gender, ethnicity, and immigration status. Discussions surfaced the need to consider and accommodate availability and accessibility needs of individuals, which may vary throughout the AI development and deployment cycle. For example, engagement may manifest at the level of *collaboration* in the IAP2 spectrum during the *define and design* stages of the AI life cycle, but for different reasons, people with lived and living experience and community may only be *involved* (IAP2 level) during the *evaluation* stage of the AI life cycle. However, it was noted that a shared bidirectional governance model between the community and the project team was central to a collaborative approach to engagement, irrespective of the AI life cycle stage.

A total of 23 workshop attendees participated in the ranking exercise with the top 6 principles (starting with the highest ranked) being: trust, equity, accountability, transparency, codesign, and value alignment.

#### Practical Considerations for Operationalizing the Key Community Engagement Principles

Participants provided insights into the practical considerations for realizing the 6 top-ranked principles to guide community engagement regarding AI deployment for addressing the burden of diabetes and other population health issues. These are in the order of ranked priority and summarized in Textbox 1.

Textbox 1. **Finalized principles of community engagement and associated practices for operationalizing each.**

**Practices**

Trust:

- Engage credible and trusted members of the community such as community leaders
- Repeated engagement with clear, honest, and consistent 2-way communication
- Demonstrated commitment to collaboration by the community and health system actors

Equity:

- Inclusiveness, diversity of community participants, and cultural responsiveness in engagement approaches
- Engagement should be responsive to the cultural and structural factors that could prevent community members from engaging in prevention practices
- Using multiple ways to engage community members in the co-design process and in the project’s governance

Accountability:

- Regular communication and feedback through community tables, gatherings, and reports
- Community coleadership in decision-making, with clear roles and responsibilities outlined
- Ability to course-correct informed by regular monitoring of the artificial intelligence (AI) tools

Transparency:

- Honest communication, declaring relevant conflicts of interest of different stakeholders, and clarity on the limitations of the AI tools
- Democratized and openly accessible platforms (eg, websites) for knowledge sharing and project updates

Codesign:

- Codesign should include diverse participants and be accessible for participation
- Continuous and iterative feedback on designing and deploying the AI tool

Value alignment:

- AI developers and implementers should understand the community’s values and priorities through meaningful dialog
- Cultural and diverse perspectives should be respected while aligning the goals of the AI tool to the community’s values

1. Trust: At least 3 concepts were described in relation to trust: building on existing relationships, giving it time, and understanding that it requires a collaborative mindset shift (in perceptions and behaviors toward each other) between the community and the health system. Participants emphasized the need for community engagement to be anchored on any existing relationships with community members and organizations who are credible, trusted by members of the community, and recognized as community leaders. Suggestions were offered on informal ways of building relationships such as spending social time together in community (eg, through community events) and fostering relationships outside specific projects or health care interactions. Discussions acknowledged that building trust on a topic as sensitive and obscure as AI would require repeated engagement with clear, honest, and consistent 2-way communication between the project team and community members. Participants expressed that if project teams failed to follow through with their commitments and responsibilities, there was a risk that whatever ground had been gained would be lost. Some additional concepts that emerged in relation to trust building were inextricably linked to the concept for accountability (ranked as the third top principle), including open and accessible communication modalities.
2. Equity: Equity was discussed from multiple perspectives, recognizing that it is not only a principle of engagement that is central to how community engagement is achieved (ie, a process measure), but is furthermore an outcome of meaningful engagement. Inclusive practices that ensure a diversity of knowledge users and representatives of people with lived and living experience, particularly in the project’s governance, were cited as an enabler for ensuring that every voice is heard. Cultural responsiveness was mentioned as a subconcept under inclusiveness and diversity. It was recommended that engagement should consider and be responsive to cultural and structural factors that make it difficult for patients to engage in preventive and promotive health behaviors. The social determinants of health were cited as shaping how community engagement on AI may address or reinforce historical and structural harm. The discussions suggested using multiple ways to engage community members in the codesign process and in the project’s governance.
3. Accountability: Accountability was linked to regular communication and feedback, project governance, and mechanisms for responsive monitoring. Some examples of avenues for regular communication and feedback at a community level included community tables, community gatherings, and through annual or biannual reports, as well as the biannual AI4DPP Solution Network meetings to share and discuss results. The ideal state was for community coleadership throughout the AI life cycle with clear roles and responsibilities by both the project team and community members, outlined in a formalized document such as a term of reference. Regular monitoring of the use of the AI tools, especially of unintended consequences, was stressed as being crucial to accountability. Feedback from workshop participants underscored the importance of a firm commitment from the project team to act on any concerns or unintended consequences that arose during AI implementation.
4. Transparency: Linked to accountability and trust, workshop participants voiced expectations of honest communication between project teams and community members with respect to knowledge sharing, and acknowledgment of the limitations of AI. For example, they suggested that users of AI need to understand the model’s limitations such as data biases. Similarly, participants noted that regular, open, and accessible communication would not only capture what works, but what is not working throughout the deployment, implementation, and evaluation phases. There were suggestions to have a project website for open dialog, feedback, and other forms of information dissemination. A participant acknowledged that the fear of failure was a disservice to the much-needed mindset of continuous improvement and learning. Establishing a community-involved governance structure was noted as an enabler for transparency and accountability.
5. Codesign: Participants proposed that codesign should include diverse participants and should consider the impact of AI on minority populations such as new immigrants and Indigenous communities. They also recommended creating a welcoming environment that promotes participation in co-designing the AI tool and its uses. To quote one of the participants, “*There aren’t any ‘points of no return,’ meaning that decisions can be reflected on and changed.*” Codesign was not perceived to be a single occurrence event, but rather, characterized by continuous and iterative feedback in AI design and deployment and throughout the life cycle.
6. Value alignment: Participants suggested that the alignment of the goals of the AI solution with community values through dialog and respect for cultural diversity was a way by which this principle could be put in action. Some participants proposed that AI developers should understand the community’s values and priorities through meaningful dialog and should use “values language.” They elaborated that any AI solution should clearly demonstrate utility in delivering benefits to the community, which could be challenging when there are diverse perspectives and cultural values within a community.

Suggestions were provided on concrete actions to guide community engagement, including the need to consider the community context, to incorporate storytelling and lived experiences from patients, and a sensitivity to knowing the right time to engage (ie, tactical engagement). Patients and people who are champions or early adopters of AI in managing their health conditions or for health promotion could strengthen community involvement by sharing compelling stories from their experiences, backed with data (eg, number of diabetes cases prevented). For project teams to demonstrate these principles, participants noted that the foundation of meaningful community engagement is building a digitally informed and literate citizenry that is empowered to contribute meaningfully to AI solutions and decisions.

## Discussion

### Principal Findings

In this study, we engaged community members, people with lived and living experience, and other health sector stakeholders to identify 6 priority principles and accompanying practices for engaging communities in population-wide deployment of AI tools, using the AI4DPP project as an example. The approach of workshop activities aligned primarily with the Involve and Collaborate levels of the IAP2 framework. Participants’ insights were reflected directly in the prioritization and operationalization of principles (Involve), and they actively influenced decision-making on which principles would be prioritized and how they would be operationalized (Collaborate). Since population level use of AI/machine learning models in health systems is geared primarily toward health care decision-makers and health care providers, our study’s findings are relevant for incorporating the missing perspective in the AI development cycle. That is, the perspectives of patients, caregivers, and other end beneficiaries. Our findings align with and extend prior research on community engagement in AI and digital health, particularly around themes of trust, diversity, transparency, and codesign. In this section, we compare our results with existing literature and reflect on their implications for health care AI implementation.

Only recently has the AI community started shifting from model-centric AI (where the accuracy of the models is the most important parameter) to data-centric AI (where the understanding of how data are collected, collated, questioned, analyzed, and interpreted is paramount) [42]. The social power that patients and their caregivers indirectly exert in shaping the sociotechnical milieu of AI adoption in health care has not been sufficiently prioritized in addressing population-level adoption of AI tools [43]. Yet, evidence shows that social acceptability of AI in health care can improve patient engagement in decision-making and enhance patients’ compliance with AI-informed care plans [44,45]. Trust was identified as the most important principle of community engagement. This is perhaps not surprising since AI has often been referred to as a “Blackbox,” increasing skepticism about its negative and unintended impact. In turn, lack of patient and public trust hinders AI implementation in health [46]. Trust is also central to how societies perceive health research and interventions, and trustworthiness is a key tenet in the ethical and responsible use of AI. There is a role, therefore, for AI researchers to open this “Blackbox” by using methods that enhance the explainability of machine learning models [47]. However, explainability from the perspective of patients and communities may differ significantly from those of technical audiences. While a full discussion of community-centered explainability is beyond the scope of this paper, we raise this point to emphasize the need for ongoing dialog on how transparency and trust in AI can be codeveloped with affected communities.

Open dialog has been reported as critical to meaningful community engagement in AI [48], and regular communication and feedback throughout a project can facilitate understanding. Patients and community partners expect honest communication about the limitations and results—positive and negative—of AI in accessible ways. They want AI researchers to commit to regularly monitor and share the results of AI implementation in health care, and to act on feedback received from community members [8]. Transparent governance structures, for example, through community advisory boards, have also been recommended to ensure accountability and build trust when involving communities on AI for health [8,49]. Because building trust takes time, necessitating ongoing engagement [48,50], it is recommended that patients and community members are engaged early, ideally at the problem identification stage and throughout the AI life cycle [51,52]. Furthermore, early engagement ensures that implementers are asking the right questions and that critical contextual factors are considered sooner than later [53]. Other researchers have also demonstrated that early and continuous involvement of community members helps to avoid tokenism [38]. Ultimately, proper alignment of researcher and community values is necessary for successful collaboration between AI researchers, developers, and communities [54].

Addressing diversity has been reported as a neglected area in patient and public involvement in health care [55,56]. Therefore, it is not surprising that inclusivity and diversity of participants in community engagement was a recurrent point raised in our study, similar to the work of others [49,51]. This is strongly related to equity, the second-ranked principle. Workshop participants stressed the need to include diverse participants across demographics, especially minorities, not only for community engagement activities but also in governance. Similarly, participants in our sessions noted that inclusivity will require culturally responsive approaches to engagement and an understanding of diverse backgrounds which may be rooted in historical and structural harm. Bringing all voices to the table can help ensure fairness and could potentially limit the perpetuation of systemic biases inherent in data and AI algorithms [47,57]. Although education was not prioritized as a standalone principle, workshop participants noted that meaningful community engagement would require a digitally informed and literate citizenry, making it nonetheless foundational for community-engaged AI interventions. The importance of AI patient education has been highlighted as crucial to meaningful community engagement and codesign [36,50].

### Strengths and Limitations

Although the starting point of our study was a rapid literature scan, which could have resulted in missing some relevant papers, the use of a participatory approach involving diverse actors including people with lived and living experience helped strengthen our findings. An additional strength of our approach is that stakeholders have now been involved early—during the pre-implementation phase of the project, which sets the stage for their contributions in other phases. Third, using a modified NGT encouraged participation and openness, elevating the perspectives of community members. Additionally, although power dynamics are often a concern in mixed-stakeholder engagement, we did not observe this. This may be due to the experience of participants with lived and living experience in patient engagement and our teams’ efforts to foster an inclusive setting before and during the workshop.

Our process has some limitations worth mentioning. While previous studies have established the significant burden of T2D in Canada [11] and Peel [24], patients and community partners were not involved in problem identification (ie, define the stage of the AI life cycle) and machine learning model development (ie, design and test stage of the AI life cycle) of the AI4DPP project. As such, community engagement was not fully embedded as conceptualized in Figure 1. Also, workshop participants represent only a small subset of the broader community—only 6 of the 30 workshop attendees were people with lived and living experience. While this limits the representativeness of perspectives reflected in this formative study, the workshop was intended as an initial step in a continual process of community engagement. Finally, using a predefined conceptual framework (Figure 1) to guide workshop discussions may have constrained the emergence of fresh ideas. Despite these limitations, our community engagement approach is a valuable contribution to the field since most existing community engagement frameworks in AI for health focus solely on the design phase of the AI life cycle [9].

### Future Directions

Building on this work, our next step is to establish a community advisory group composed of people with lived and living experience and community organization representatives. This group will support the implementation and evaluation phases of the AI4DPP project, providing feedback on how well the principles and practices identified in this study are being enacted. This will inform the development of community-informed guidelines to support AI deployment in public health. The identified principles and practices are early inputs for a community engagement framework to deploy AI prediction tools for population health, which will be refined and tested in subsequent phases of the AI4DPP project. Future research could further refine participatory strategies and embed longitudinal engagement mechanisms throughout the AI life cycle.

### Conclusions

This study described a formative qualitative process used to identify principles and practices for meaningful community engagement in the deployment of AI tools for diabetes prevention and management. Using the context of Peel region, these insights reflect the priorities and expectations of community members, patients, and public health stakeholders. While the outputs are specific to our study context, they may be useful to others designing or adapting engagement strategies for AI implementation in public health settings. Our findings offer practical considerations for researchers, AI developers, clinicians, and community partners seeking to embed equity-oriented engagement practices into the design and deployment of AI-driven interventions.

## Abbreviations

AI: artificial intelligence
AI4DPP: Artificial Intelligence for Diabetes Prediction and Prevention
PFP: Patient and Family Partners
T2D: type 2 diabetes
